# MOGAD Is the Most Common Cause of Isolated Optic Neuritis in Children

**DOI:** 10.1002/acn3.70422

**Published:** 2026-05-04

**Authors:** Chaitanya Aduru, Akansha Chandrasekar, Kyla Blasingame, Jonathan Rosen, Jesse M. Levine, Karla Salazar, Madhuri Chilakapati, Rod Foroozan, Victoria Hardwick, Jonathan M. Yarimi, Nikita Shukla, Timothy E. Lotze, Kristen S. Fisher, Alexander J. Sandweiss

**Affiliations:** ^1^ Department of Pediatrics, Section of Neurology and Developmental Neuroscience Baylor College of Medicine and Texas Children's Hospital Houston Texas USA; ^2^ Department of Ophthalmology Baylor College of Medicine and Texas Children's Hospital Houston Texas USA

**Keywords:** demyelination, multiple sclerosis, neuroinflammatory, optic nerve, pediatric neurology

## Abstract

**Objectives:**

The study aimed to characterize the clinical features, etiologies, and outcomes of isolated, first‐time pediatric ON in the post‐MOG‐IgG era.

**Methods:**

This was a single‐center retrospective cohort study at Texas Children's Hospital of patients diagnosed with first‐time ON between 2018–2024, with follow‐up data collected through 2025. Inclusion criteria required monocular or binocular subacute vision loss with supportive paraclinical signs. Subjects were excluded if they had a prior history of demyelinating disease or extra‐orbital demyelinating lesions on initial brain/spine MRI. Primary outcomes were the proportion of MOG‐IgG seropositivity, retinal nerve fiber layer thickness, and visual acuity at onset and most recent follow‐up.

**Results:**

Of 73 children with ON, 38 met criteria for isolated first‐time ON. Etiologies included MOGAD (*n* = 27, 71.1%) and idiopathic/monophasic (*n* = 9, 23.7%), while none of the 38 subjects were later diagnosed with multiple sclerosis. MOGAD‐ON was associated with less severe vision loss at presentation (*p* < 0.01) compared to idiopathic cases. At most recent follow‐up, both groups achieved excellent functional recovery, yet both demonstrated significant retinal nerve fiber layer thinning from ON presentation. Linear regression revealed worse presenting LogMAR significantly correlated to thinner follow‐up RNFL (F(1,18) = 8.467, R^2^ = 0.32, *p* < 0.01). One (3.7%) MOGAD‐ON patient relapsed during follow‐up.

**Interpretation:**

In isolated pediatric ON, no patients were diagnosed with MS, and MOGAD is the predominant etiology. The linear correlation between initial LogMAR and subsequent RNFL atrophy suggests a permanent reduction in neurological reserve dependent on the degree of functional severity at ON onset.

## 1 Introduction

Optic neuritis (ON) is an inflammatory condition of the optic nerve commonly associated with central nervous system (CNS) demyelinating disease, among other autoimmune, infectious, and idiopathic etiologies [1]. Initial presentation of ON varies greatly, but common symptoms include loss of visual acuity, retro‐orbital eye pain, reduced color vision, and relative afferent pupillary defect (RAPD) [2]. Diagnosis of ON is crucial as long‐term visual prognosis varies based on etiology, including differences between specific CNS demyelinating diseases [3]. High‐dose intravenous methylprednisolone (IVMP) is the mainstay of acute treatment, which accelerates recovery but differs in its effect on long‐term visual outcomes by ON etiology [4, 5, 6, 7]. Although ON is well‐characterized in adults, immune‐mediated mechanisms and myelination patterns differ in children, necessitating research on pediatric ON. Even with severe initial vision loss, most children recover with good long‐term outcomes [8, 9].

Myelin oligodendrocyte glycoprotein (MOG) antibody‐associated disease (MOGAD) is an inflammatory demyelinating disorder of the CNS that accounts for approximately 20%–30% of pediatric inflammatory CNS syndromes [10, 11]. The most frequent clinical manifestation is ON, with additional phenotypes including acute disseminated encephalomyelitis (ADEM), transverse myelitis, cerebral cortical encephalitis (CCE), and brainstem or cerebellar syndromes [10, 12, 13]. The diagnosis of MOGAD requires the presence of MOG‐IgG in the serum or rarely in the CSF [14], in addition to supportive clinical and radiographic features. Access to cell‐based assays for the detection of MOG‐IgG has increased over the past decade, thereby improving diagnostic yield [15].

While other CNS demyelinating disorders such as multiple sclerosis (MS) or neuromyelitis optica spectrum disorder (NMOSD) may also present with ON, their clinical trajectories and relapse rates differ from MOGAD [16]. Notably, most subjects included in investigations of pediatric ON likely predate the routine inclusion of MOG‐IgG testing in diagnostic evaluations. Therefore, we sought to characterize the etiologies of pediatric ON at initial presentation, with particular emphasis on isolated ON (i.e., absence of additional CNS lesions consistent with a syndromic demyelinating disorder). We report here a single‐center retrospective study of isolated first‐time pediatric ON.

## 2 Methods

### 2.1 Study Design and Patient Population

This is a single‐center retrospective analysis of ON at Texas Children's Hospital in Houston, TX, USA diagnosed between 2018–2024 (when serum MOG‐IgG was commercially available to test), with follow‐up data collected through 2025. Patients were identified in the hospital or in the outpatient neurology or ophthalmology clinics to have ON by clinical investigators (MC, RF, VA, JMY, NS, TEL, KFS, and AJS) as part of an ongoing research protocol and cross‐referenced through a search of the institutional electronic medical record using ICD‐10 codes for optic neuritis (H46.X). Subjects were then filtered as determined by inclusion criteria previously described [2] per the international diagnostic criteria for ON, summarized as: monocular or binocular subacute loss of vision associated with reduced contrast and color vision, an RAPD (if the ON is unilateral or asymmetric), with or without orbital pain on eye movement, or with supportive paraclinical signs, including contrast enhancement of the optic nerve on MRI, optic disc swelling on optical coherence tomography (OCT), or a known biomarker of disease (i.e., MOG‐IgG, AqP4‐IgG, or CSF oligoclonal bands). Patients were excluded if they had a prior history of a demyelinating condition or were found to have additional demyelinating lesions outside of the optic nerve(s) on initial MRI. Of the remaining patients with only optic nerve involvement, those who were eventually diagnosed with an alternative condition (i.e., ON mimickers) and those who did not undergo serum MOG‐IgG testing were also excluded. Patients were then assessed for relapse, defined as a new demyelinating presentation > 30 days after the initial insult.

### 2.2 Extracted Variables

Cerebrospinal fluid (CSF) and serum antibody studies were evaluated via cell‐based assay at either Mayo Clinic Laboratories (Rochester, MN), ARUP Labs (Salt Lake City, UT), or both. Clinical information, demographics, labs, and imaging were retrospectively obtained from the electronic medical records of each patient.

Data are presented as percent positive for dichotomous variables and as the median and range for latencies to events and ages. Continuous numerical variables with a normal distribution are described as the average ± standard error of the mean (SEM), and outliers greater than 2 standard deviations above the mean were excluded. CSF pleocytosis was defined as a CSF white blood cell (WBC) count greater than 5 cells/mm^3^, and antibody (Ab) titers were log‐transformed using the formula *y* = log_2_(*x*) + 1 such that the transformation reflects the “dilution factor” of the titer (i.e., 1:1 = 1, 1:2 = 2, 1:4 = 3, 1:8 = 4, etc.) [17, 18]. Visual acuity was converted to logarithm of minimum angle of resolution (LogMAR) and compared between presentation and most recent follow‐up.

### 2.3 Statistical Analysis

Chi‐squared statistic was employed for dichotomous variable comparisons, and Fisher's exact test was used for smaller group sizes (*n* < 5). Mann–Whitney U test was employed to compare two groups of ordinal values or continuous numerical values with non‐parametric distributions. Kruskal‐Wallis test was used to compare more than two groups of either ordinal values or non‐parametric continuous numerical values, and Two‐Way ANOVA was used to compare the interaction between more than two groups across time. Linear regression was used to compare the relationship between two continuous variables and logistic regression for comparing binary classifications. Comparisons were considered statistically significant at *p* < 0.05. Statistical analyses were performed with GraphPad Prism 11.0 (GraphPad Software, Boston, Massachusetts USA).

### 2.4 Study Approval

The study was approved by the Institutional Review Board at Baylor College of Medicine and Texas Children's Hospital, Houston, TX.

## 3 Results

### 3.1 Proportion of Total ON Cases

**FIGURE 1:**
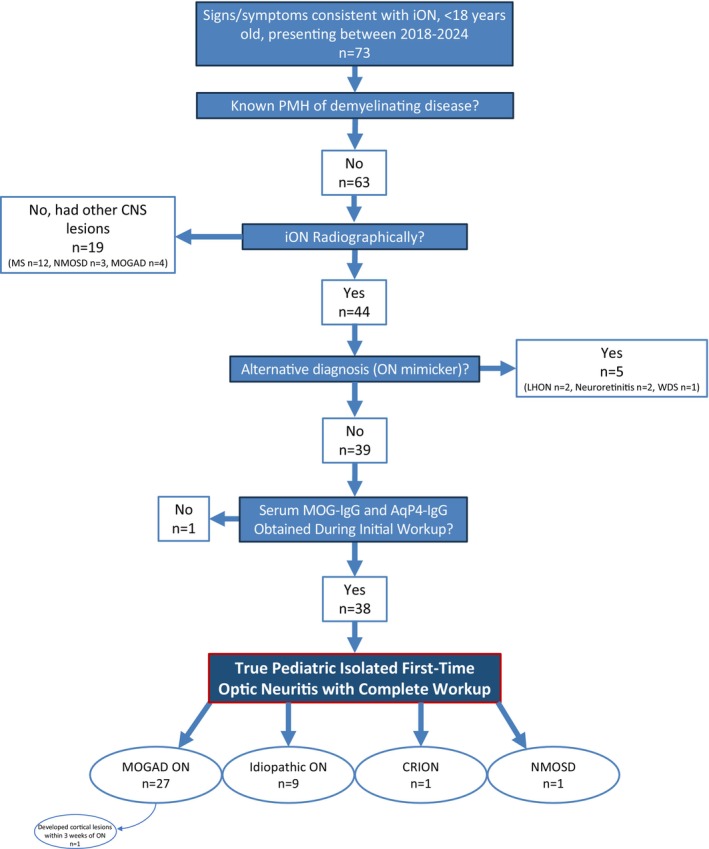
Flow diagram of study design. Abbreviations: CNS, central nervous system; CRION, chronic relapsing inflammatory optic neuropathy; iON, isolated optic neuritis; LHON, Leber hereditary optic neuropathy; MOGAD, myelin oligodendrocyte glycoprotein antibody disorder; NMOSD, neuromyelitis optica spectrum disorder; PMH, past medical history; WDS, white dot syndrome.

There were 73 children with ON of any kind from 2018 to 2024 (Figure 1). 63/73 had no prior medical history of a demyelinating condition (i.e., the other 10 were already previously diagnosed with NMOSD, MOGAD, or MS). Of the 63 patients with no relevant medical history (i.e., presenting with first‐time ON), 19 were evident on initial MR imaging to have *additional* demyelinating lesions and were ultimately diagnosed with a known syndromic etiology (MS *n* = 12, NMOSD *n* = 3, MOGAD *n* = 4). Of the remaining 44 patients with isolated ON, 5 were ultimately diagnosed with an alternative diagnosis (Leber hereditary optic neuropathy *n* = 2, neuroretinitis *n* = 2, white dot syndrome *n* = 1). 38/39 remaining patients had serum MOG‐IgG testing as part of their initial workup. All 38 remaining patients had MR brain and orbits, and 28/38 also had spinal imaging (25/28 included complete spine). Of the patients with isolated ON, 27 were MOG‐IgG seropositive (1/27 with a live‐cell titer of 1:50, the rest at least 1:200), 1 had serum AqP4‐IgG (NMOSD), 1 had a clinical course consistent with chronic inflammatory optic neuropathy (CRION), and 9 were idiopathic and monophasic during the study period through 2025. Because the vast majority of isolated ON cases were either MOGAD‐ON (*n* = 27) or Idiopathic ON (*n* = 9), the remainder of the study focuses on these 36 cases.

### 3.2 Demographics and Clinical Features of MOGAD‐ON vs. Idiopathic ON

Both groups had a similar median age at symptom onset (MOGAD‐ON: 12 [4.5–17] years vs. Idiopathic ON: 12 [4.7–17] years, *p* = 0.74) and a comparable sex distribution (female patients in MOGAD‐ON: 14/27 (51.9%) vs. Idiopathic ON: 6/9 (66.7%), *p* = 0.44). There was no difference by self‐identified race (roughly similar distribution of white, black, and Asian) or self‐identified Hispanic ethnicity between the groups (MOGAD‐ON: 18/27 (66.7%) vs. Idiopathic ON patients: 4/9 (44.4%), *p* = 0.27, Table 1).

**TABLE 1:** Demographics and Clinical Characteristics of patients with MOGAD‐ON and Idiopathic ON.

	MOGAD	Idiopathic	*p*
Age (years) (median [range])	12 [4.5–17]	12 [4.7–17]	0.74
Sex (female)	14/27 (52%)	6/9 (67%)	0.44
Race			0.70
White Black Asian	23/27 (85.2%) 3/27 (11.1%) 1/27 (3.7%)	7/9 (77.8%) 1/9 (11.1%) 1/9 (11.1%)	
Ethnicity (Hispanic)	18/27 (67%)	4/9 (44%)	0.27
Bilateral eye involvement	12/27 (44%)	3/9 (33%)	0.71
Unilateral eye involvement (left)	8/15 (53%)	3/6 (50%)	0.99
Orbital pain	23/27 (85%)	4/9 (44%)	< 0.05*
Latency to presentation (days) (median [range])	6.0 [1–23]	4 [2–22]	0.66
CSF WBC (cells/mm^3^)	10.3 ± 2.1	4.7 ± 2.2	0.08
CSF pleocytosis	11/17 (65%)	2/6 (33%)	0.18
CSF protein (mg/dL)	40.5 ± 7.4	28.5 ± 4.2	0.33
Vitamin D (ng/mL)	20.5 ± 1.5	23.9 ± 5.6	0.40
EBV serologies			0.99
Recent infection Remote infection No prior infection	2/13 (15.4%) 7/13 (53.8) 4/13 (30.8%)	0/5 (0%) 4/5 (80.0%) 1/5 (20.0%)	
Number of segments			0.43
1 segment 2 segments 3 segments	7/27 (26%) 11/27 (41%) 9/27 (33%)	0/9 (0%) 6/9 (67%) 3/9 (33%)	
Intraorbital	25/27 (93%)	8/9 (89%)	> 0.99
Intracanalicular	21/27 (78%)	9/9 (100%)	0.22
Intracranial	10/27 (37%)	4/9 (44%)	0.71
Initial mean RNFL thickness (um)	167.8 ± 58.02	207.1 ± 71.28	0.13
Final mean RNFL thickness (um)	78.4 ± 4.0	81.0 ± 7.6	0.77
Initial mean LogMAR	0.81 ± 0.16	1.69 ± 0.16	< 0.01*
Final mean LogMAR	0.13 ± 0.06	0.39 ± 0.25	0.52
Follow‐up duration (months); median (IQR)	19.6 (6.5–33.8)	11.9 (1.5–27.1)	0.41

Abbreviations: CSF, cerebrospinal fluid; EBV, Epstein‐Barr virus; MOGAD‐ON, myelin oligodendrocyte glycoprotein antibody disorder optic neuritis; RNFL, retinal nerve fiber layer; WBC, white blood cell.

* *p* < 0.05.

Bilateral eye involvement was observed in 12/27 (44.4%) of the MOGAD‐ON group and in 3/9 (33.3%) of the Idiopathic ON group (*p* = 0.71). There was no difference in sidedness among patients with unilateral ON (left eye involvement in MOGAD‐ON: 8/15 (53.3%) vs. Idiopathic ON: 3/6 (50%), *p* = 0.99). Orbital pain as part of the chief complaint was reported significantly more often in the MOGAD‐ON group (23/27, 85.2%) compared to the Idiopathic ON group (4/9, 44%, *p* < 0.05). There was no difference in latency to presentation after symptom onset between groups (MOGAD‐ON: 6.0 days [1–23] vs. Idiopathic ON: 4.0 days [2–22], *p* = 0.66).

### 3.3 CSF And Serologies of MOGAD‐ON vs. Idiopathic ON

CSF studies were collected for 17/27 patients in the MOGAD‐ON group and 6/9 patients in the Idiopathic ON group. There was no difference in CSF WBC count between MOGAD‐ON and Idiopathic ON (10.3 ± 2.1 cells/mm^3^ vs. 4.7 ± 2.2 cells/mm^3^, respectively, *p* = 0.08), nor was there a difference in the proportion of subjects with CSF pleocytosis (MOGAD‐ON: 11/17 (65%) vs. Idiopathic ON: 2/6 (33%), *p* = 0.18). There was no difference in CSF protein between those with MOGAD‐ON and Idiopathic ON (40.5 ± 7.4 mg/dL vs. 28.5 ± 4.2 mg/dL, respectively, *p* = 0.33).

Serum Vitamin D level was collected in a limited number of patients, as deemed clinically relevant by the care team. Serum vitamin D was not different between the 17/27 patients in the MOGAD‐ON group and 5/9 in the Idiopathic ON group in whom it was collected (20.5 ± 1.5 ng/mL vs. 23.9 ± 5.6 ng/mL, respectively, *p* = 0.40). Because EBV has been implicated in some cases of MOGAD [19], we also evaluated EBV serologies between groups and found no difference in the proportion of recent vs. remote EBV infection or no prior EBV infection (MOGAD‐ON: 2/13 with recent infection, 7/13 with remote infection, 4/13 never infected, with 1 inconclusive result vs. Idiopathic ON: 4/5 tested with remote infection and 1/5 never infected).

### 3.4 Optic Nerve Focality of MOGAD‐ON vs. Idiopathic ON

The optic nerve focality on MRI imaging, described as either intraorbital, intracanalicular, and/or intracranial, was compared between the MOGAD‐ON group and the Idiopathic ON group. There was no difference in the number of optic nerve segments involved between the two groups. 7/27 (25.9%) in the MOGAD‐ON group had only one segment involved compared to 0/9 (0%) in the Idiopathic ON group, while 11/27 (40.7%) in the MOGAD‐ON group had two segments involved compared to 6/9 (67%) in the Idiopathic ON group, and 9/27 (33.3%) of the MOGAD‐ON group had involvement of all three segments compared to 3/9 (33%) in the Idiopathic ON group. There was no difference in which optic nerve segments were involved by etiology. 25/27 (92.6%) of the MOGAD‐ON group and 8/9 (89%) of the Idiopathic ON group involved the intraorbital segment (*p* > 0.99), 21/27 (77.7%) of the MOGAD‐ON group and 9/9 (100%) of the Idiopathic ON group involved the intracanalicular segment (*p* = 0.22), and 10/27 (37.0%) of the MOGAD‐ON group and 4/9 (44%) of the Idiopathic ON group involved the intracranial segment (*p* = 0.71).

### 3.5 Treatments, Oral Steroid Taper, and Maintenance Therapy

#### 3.5.1 Acute Treatment

17/27 (63.0%) patients with MOGAD‐ON received IVMP alone and 10/27 (37.0%) received IVMP + IVIG at presentation with median latency from symptom onset to steroids 8 days (1–38 days). 7/9 (77.8%) patients with Idiopathic ON received IVMP only, 1/9 (11.1%) received IVMP + IVIG, and 1 patient refused IV treatment (and therefore received high dose oral steroids) with median latency to steroids 6 days (3–23 days). 5/27 (18.5%) patients with MOGAD‐ON also received plasma exchange (PLEX) therapy during the acute hospitalization for optic neuritis—due to severity of visual symptoms or lack of response to other immunotherapy, per the discretion of the treating physician—while none of the patients with Idiopathic ON received PLEX. We observed a difference in how often IVIG was administered during the course of the study: between 2018–2021 1/7 (14.3%) of those with MOGAD‐ON received IVIG while between 2022–2024 9/20 (45.0%) received IVIG.

### 3.6 Oral Steroid Taper

Most patients with either MOGAD‐ or Idiopathic ON were given an oral steroid taper of prednisone lasting days to weeks. The most common starting dose of prednisone was 60 mg (24/34 patients who were given an oral steroid taper started at 60 mg). 26/27 patients with MOGAD‐ON received an oral steroid taper following their acute hospitalization for ON (median duration 42 days, range 15–49 days, IQR 34–42 days). 20/26 (76.9%) were on the oral steroid taper for 5+ weeks while 6/26 (23.1%) were on it for < 5 weeks. 8/9 patients with Idiopathic ON received an oral steroid taper following their acute hospitalization (median duration 31.5 days, range 4–42 days, IQR 16.5–42 days). 4/8 were on an oral steroid taper for 5+ weeks while 4/8 were on it for < 5 weeks.

### 3.7 Maintenance IVIG

5/27 (18.5%) patients with MOGAD‐ON were treated with monthly IVIG for at least 6 months following their acute hospitalization with ON (1 of the 5 patients who also received PLEX during the acute hospitalization) while none of those with Idiopathic ON were treated with maintenance therapy.

### 3.8 RNFL Thickness and Visual Acuity Over Time of MOGAD‐ON vs. Idiopathic ON

**FIGURE 2:**
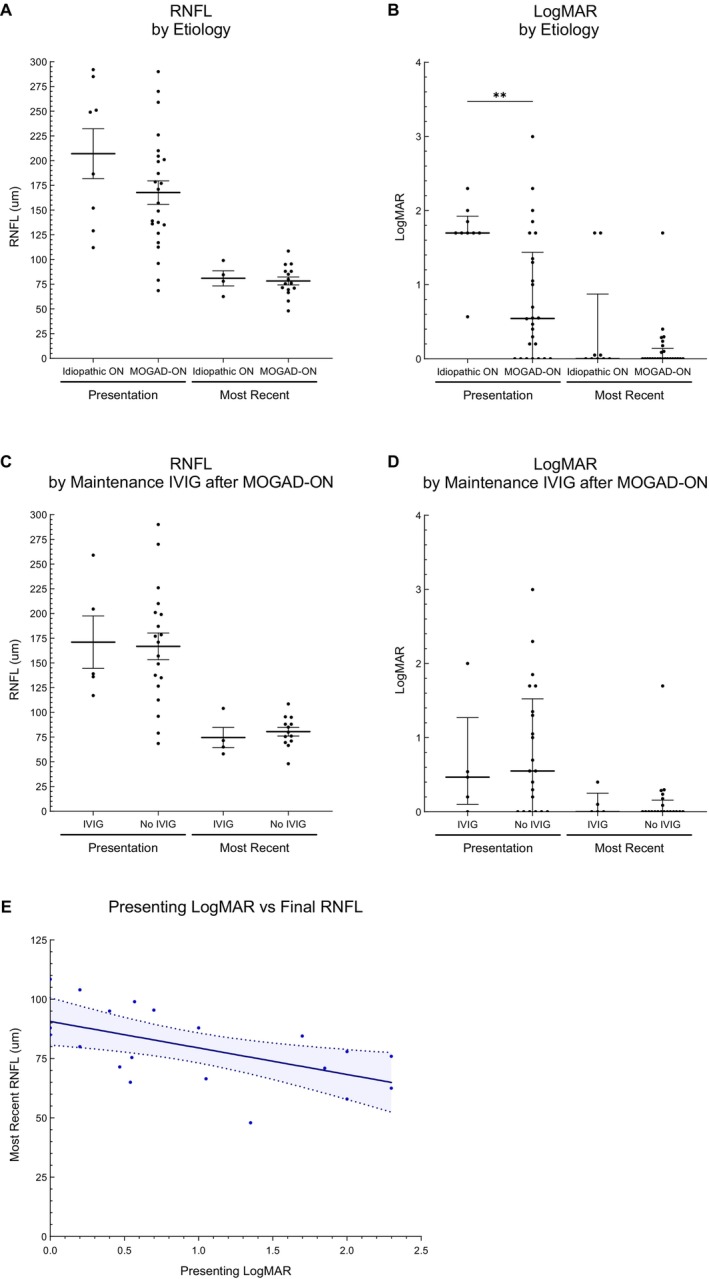
Retinal Nerve Fiber Layer Thickness and Visual Acuity at Optic Neuritis Presentation and Follow‐Up. (A) There was no difference in RNFL between those with Idiopathic ON and MOGAD‐ON either at presentation or most recent follow‐up. (B) LogMAR was significantly higher at presentation in those with Idiopathic ON compared to MOGAD‐ON (1.69 ± 0.16 vs. 0.81 ± 0.17, respectively; *p* < 0.01), but there was no difference at most recent follow‐up. There was no difference in either (C) RNFL or (D) LogMAR at presentation or most recent follow‐up between those that received maintenance IVIG and those that did not. (E) There is a statistically significant simple linear relationship between LogMAR at presentation and RNFL at most recent follow‐up (F(1,18) = 8.467, r^2^ = 0.32, *p* < 0.01, *n* = 20). Abbreviations: IVIG, intravenous immunoglobulin; LogMAR, logarithm of the minimum angle of resolution; MOGAD‐ON, myelin oligodendrocyte glycoprotein antibody associated disease optic neuritis; RNFL, retinal nerve fiber layer thickness.

Differences in retinal nerve fiber layer (RNFL) thickness and visual acuity were measured at presentation and over time between the MOGAD‐ON and Idiopathic ON groups. At presentation, there was no significant difference in mean RNFL thickness between the MOGAD‐ON and Idiopathic ON groups (167.8 ± 58.02 um and 207.1 ± 71.28 um, respectively, *p* = 0.13), and there was no difference in RNFL thickness between groups at most recent follow‐up either (78.4 ± 4.0 um and 81.0 ± 7.6 um, respectively, *p* = 0.77; Figure 2A). LogMAR was significantly lower at presentation in those with MOGAD‐ON compared to those with Idiopathic ON (0.81 ± 0.16 vs. 1.69 ± 0.16, respectively, *p* < 0.01), but there was no difference in visual acuity between groups at most recent follow‐up (0.13 ± 0.06 and 0.39 ± 0.25, respectively, *p* = 0.52; Figure 2B) with a median follow‐up duration of 19.6 (IQR: 6.5–33.8) months in the MOGAD‐ON group vs. 11.9 (IQR: 1.5–27.1) months in the Idiopathic ON group (*p* = 0.41).

There was neither a difference in RNFL at presentation nor at 12 to 18‐month follow‐up between those who received maintenance IVIG and those who did not (presentation RNFL: Maintenance IVIG 171.1 ± 23.7 um vs. No IVIG 160.2 ± 14.1 um, *p* = 0.73; 12 to 18‐month RNFL: Maintenance IVIG 74.6 ± 8.8 um vs. No IVIG 80.5 ± 4.1 um, *p* = 0.62; Figure 2C). Additionally, there was neither a difference in LogMAR at presentation nor at 12 to 18‐month follow‐up between those who received maintenance IVIG and those who did not (presentation LogMAR: Maintenance IVIG 0.64 ± 0.32 vs. No IVIG 0.85 ± 0.19, *p* = 0.61; 12 to 18‐month LogMAR: Maintenance IVIG 0.1 ± 0.07 vs. No IVIG 0.14 ± 0.08, *p* = 0.73; Figure 2D).

Among the combined cohort of MOGAD‐ON and Idiopathic ON, visual acuity at acute presentation of ON had no correlation to eventual recovery visual acuity at most recent follow‐up, nor did RNFL thickness at presentation correlate to RNFL thickness at most recent follow‐up. However, simple linear regression revealed a significant inverse linear relationship between LogMAR at presentation and most recent RNFL thickness, i.e., greater LogMAR at presentation (worse visual acuity) correlated to thinner RNFL at most recent follow‐up (F(1,18) = 8.467, r^2^ = 0.32, *p* < 0.01, *n* = 20; Figure 2E).

### 3.9 MOGAD‐ON Relapses

1/27 patients with isolated MOGAD‐ON (3.7%) relapsed after their initial ON presentation with median follow‐up duration of 19.6 months, IQR 6.5–33.8 months versus median 11.9 months, IQR 1.5–27.1 months in the Idiopathic‐ON group (*p* = 0.41). The patient was a 5‐year‐old female with bilateral ON. She received IVMP with rapid improvement in her vision. She did well with a LogMAR of 0 for nearly 5 years until she relapsed with bilateral ON in addition to new involvement of the brainstem and bilateral hypothalamus and a positive repeat serum MOG‐IgG. She acutely received IVMP and IVIG followed by monthly IVIG thereafter with eventual recovery of her vision. She was still on monthly immunoglobulin and had LogMAR of 0.0 (20/20 vision) with 69 um and 74 um RNFL thickness of her right and left eyes, respectively. There remain subtle T2 hyperintensities of the brain structures affected during the relapse.

## 4 Discussion

This is the largest single‐center retrospective pediatric case series of first‐time isolated ON. We observed several key clinically relevant outcomes: (1) when we filtered for truly isolated first‐time ON, none of the patients were ultimately diagnosed with MS. (2) More than two‐thirds of patients presenting with isolated ON were diagnosed with MOGAD while most of the remaining patients were diagnosed with both idiopathic and monophasic (as of this writing) ON. (3) There is an inverse linear relationship between severity of visual acuity deficit at ON presentation and RNFL of the affected eye months later (we provisionally hypothesize the degree of acute vision loss—even with complete recovery—may risk visual impairment in adulthood). (4) Only 1 of 27 MOGAD‐ON patients later relapsed (as of this writing), suggesting truly isolated MOGAD‐ON rarely recurs in pediatric populations.

### 4.1 Isolated Optic Neuritis Did Not Develop Into Multiple Sclerosis

Corroborating prior research from the “pre‐MOGAD” era, children who present with isolated optic neuritis and have no other CNS demyelinating lesions did not develop MS during the observation time in our study [9]. This differs from some adult studies which report a variable 14%–42% risk of developing MS within several years of isolated ON without other CNS lesions [20, 21, 22, 23]. Importantly, this contrasts with those pediatric patients who presented clinically with only ON—and had no prior pertinent medical history—but had other silent radiographic CNS lesions suggestive of a demyelinating syndrome such as MS, NMOSD, or MOGAD. Our data suggest about one‐quarter of the children in our cohort who presented with isolated ON ultimately met 2024 McDonald Criteria, all of whom also had other CNS lesions. This corroborates past pediatric studies that found a similar rate of children (about one‐fifth or more) who presented with an index event of ON with silent radiographic CNS lesions on MRI who ultimately met McDonald Criteria (various iterations of the criteria depending on the date of the study) for the diagnosis of MS [23, 24, 25]. It is important to note that a myriad of prior studies evaluating pediatric isolated optic neuritis were published in the “pre‐MOGAD” era; many of the patients who were identified as ‘Idiopathic ON’ may have had MOGAD‐ON.

### 4.2 Most Isolated Optic Neuritis was Secondary to MOGAD

Our study found two‐thirds of the pediatric patients with isolated ON and no other CNS demyelinating lesions were ultimately diagnosed with MOGAD, considerably more than we hypothesized. While there are plenty of other studies on pediatric ON that also stratified the ultimate diagnoses or triggers of ON [26, 27], our study is novel as it focused specifically on patients after the emergence of universally available MOG‐IgG testing without a prior medical history who presented with truly isolated ON (without radiographic lesions), a common clinical conundrum neurologists face when discussing potential etiologies and risk of relapse with families and caregivers. While other studies—including those with long‐term follow‐up—identified between 29%–54% of pediatric patients with ON to be diagnosed with MOGAD, the exclusion criteria for prior studies and ours are subtly different, and thus we answered different, albeit all clinically relevant, questions [23, 26, 28]. In other words, the denominator in each of these studies includes heterogeneous cohorts. For example, some earlier cohorts did not exclude patients who did not have MOG‐IgG testing completed (as this likely would have left too few patients for analyses), so patients that had MOG‐IgG testing completed may have introduced a selection bias toward those more likely to have MOGAD. Our study cohort includes patients diagnosed as early as 2018, when standard workup for ON (or other clinically applicable phenotypes) included testing for MOG‐IgG at our institution. The high proportion of MOGAD‐ON in our cohort reinforces the necessity of universal MOG‐IgG testing in pediatric patients presenting with isolated ON, as idiopathic cases may now be in the minority.

### 4.3 Differentiators of MOGAD‐ON vs. Idiopathic ON

The affected segment of optic nerve provided no predictive capacity for differentiating MOGAD‐ON from Idiopathic ON in our cohort of isolated ON. However, our findings are consistent with prior multi‐center and international studies in that MOGAD‐ON tended to affect the anterior segment of the optic nerve more often than not [29, 30, 31]. This consistent finding is despite key differences in study methodology compared to prior case series, including our exclusion criteria of patients with extra‐orbital CNS lesions, and most other studies included adults. Additionally, our findings are consistent with prior characterizations of longitudinally extensive lesions of the optic nerve in MOGAD‐ON, classically defined as involvement of two or more contiguous optic nerve segments. Three‐quarters of our MOGAD‐ON cohort involved two or more optic nerve segments, but all of the Idiopathic ON patients had longitudinally extensive lesions too. There was no predictive value of either location or length of optic nerve involvement in stratifying etiology between MOGAD‐ON and Idiopathic ON, unlike the predictive value in differentiating from NMOSD and MS [29]. While orbital pain is a common presenting symptom in children with optic neuritis, the presence of orbital pain was significantly more frequent in those with MOGAD‐ON than idiopathic. MOGAD‐ON is hypothesized to cause considerable perineural inflammation and edema (variables we did not measure in this study), which may contribute to the development of orbital pain [32, 33]. The presence of orbital pain with eye movements in a child with acutely changing visual acuity should warrant prompt consideration of MOGAD‐ON.

### 4.4 Complete Recovery of Visual Acuity and Implications for Adulthood Vision

Interestingly, we found that while RNFL thickness at acute presentation did not differ by etiology, visual acuity was worse in those with Idiopathic ON than MOGAD‐ON, perhaps lending itself as a potential predictive tool in stratifying etiology. We wondered if this could be explained by those with MOGAD‐ON presenting to healthcare earlier than those with Idiopathic ON, perhaps due to the presence of concomitant orbital pain, and thus acquired less‐severe visual acuity loss. However, there was no difference in latency to presentation; it seems that the underlying mechanism of disease is the most likely explanation for varied visual acuity rather than time‐to‐vision check alone. MOGAD‐ON is often described as devastating vision loss, and indeed we had several children with the equivalent of LogMAR 1.3 or higher (20/400 vision or worse), and in some cases could only count fingers or had no visual light perception. But more than one‐third of children presented with unaffected visual acuity or only mild loss of visual acuity. Many of these patients presented with either orbital pain or color desaturation as the impetus for optic neuritis workup. This wide variability in visual acuity at presentation may represent the heterogenous nature of MOGAD‐ON. It is also important to consider the difference in exclusion criteria in our study vs. prior reports that often include patients with more than just isolated MOGAD‐ON (i.e., with other simultaneous extra‐orbital CNS lesions). At most recent follow‐up, there was no difference in either RNFL thickness or visual acuity between the two etiologies, although retinal ganglion layer (GCL) thickness, which was unavailable in this retrospective study, may be a more specific surrogate marker of visual acuity and long‐term outcomes [34, 35]. Nearly three‐quarters of the children in this study recovered their visual acuity to at least the equivalent of 20/40 vision or better (LogMAR 0.3 or lower), with many recovering to 20/20 (LogMAR 0.0), though it is important to remember some of the patients with MOGAD‐ON had unaffected visual acuity to begin with. Visual acuity recovery occurred despite the significant decrease in RNFL thickness months after acute ON, though we cannot say how this new RNFL thickness compares to their pre‐ON baseline. We can, however, compare our cohort's RNFL thickness to expected norms, which fall within the 90–110 μm thickness for healthy subjects under 20 years old and decrease by 0.16 to 0.44 μm/year thereafter [36, 37, 38, 39]. The RNFL in most children in our cohort fell below that range months after their bout of ON. Despite this, complete visual acuity recovery is common in the first 6 months [23, 28], which our results corroborate. Visual acuity worsens during normal aging‐related optic nerve degeneration, but there is seemingly a buffer of 10–20 μm [40, 41, 42]. It is unknown how childhood optic neuritis affects adulthood visual acuity years later and there are currently no published studies examining the effect of childhood ON on adulthood vision. But with the abrupt decrease in RNFL thickness after ON, we provisionally hypothesize that because of this neurological reserve, pediatric ON establishes a new “curve” and may accelerate age‐related visual acuity decline in adulthood, a hypothesis requiring a multiple decades'‐long prospective study into adulthood or a cross‐sectional study of adults who had pediatric ON.

### 4.5 Relapse Rate

The reported rate of relapse in MOGAD varies widely by study type and specificity of the cohort explored [32, 43, 44, 45, 46]. Our cohort of isolated first‐time ON had one patient relapse, a patient with MOGAD‐ON. They underwent a very similar clinical course during their initial bout of ON as the other subjects in the cohort and their visual acuity resolved. Still, they relapsed with extra‐orbital involvement of the brainstem and bilateral hypothalamus 5 years after the incident event. We could not identify any risk factors or differences in management that predicted their relapse, except perhaps the absence of IVIG at acute presentation. Though most prior research reports the highest probability of relapse after ON is within the first 2 years [47, 48], our patient relapsed outside that window. We have not detected any other relapses in the cohort, but there are major limitations that our retrospective study design introduces, including the lack of consistent standardized follow‐up. Subjects followed up in either neurology or ophthalmology clinic (usually both) for nearly 2 years, but some patients did not follow up at all with our group or only followed up for a short period of time. We therefore cannot conclude the relapse rate in isolated pediatric MOGAD‐ON is necessarily as low as our data suggest alone, but there are key differences between our study and prior studies that suggest a much higher relapse rate. Notably, many prior studies on MOGAD outcomes did not differentiate a core clinical event of isolated ON vs. ON with extra‐orbital CNS lesions as we did in our study. While the seminal article by Nosadini et al. ultimately found that the presence of abnormal optic nerves on MRI during an incident MOGAD presentation was protective against relapse, the rate of ON‐related relapses was still 8/28 (28.6%) among those with ON in their MOGAD cohort [44], much higher than ours. Their study had a considerably longer duration of follow‐up, a median 30 months compared to 19 months in ours. We may have simply missed relapses in our single‐center study or have not followed up long enough to detect the patients, since we only include subjects with ON dating back to 2018 when we began testing for MOG‐IgG routinely (whereas Nosadini et al. collect data dating back to 2016). Further, they found early immunotherapy and long oral prednisone tapers were associated with lower risk of relapsing disease with median time to immunotherapy 5.5 days in their all‐phenotype MOGAD cohort. Median time to initial immunotherapy was 8.0 days in our isolated optic neuritis cohort and half the subjects received immunotherapy within 7 days of symptom onset, though it is unclear how this compares to their optic neuritis group. Finally, our group was influenced by Nosadini and other contemporary studies reporting the effects of early immunotherapy and/or IVIG [49, 50], which likely altered our practice patterns, leading to increased rate of concomitant IVIG administration with IVMP. We therefore had a greater proportion receiving IVIG at the incident event (more than one‐third in our study compared to less than one‐fifth in the Nosadini et al. study). All of our patients with MOGAD‐ON received IVMP and a greater proportion of our cohort received an oral steroid taper for 5 or more weeks, which may have also reduced the odds of relapse in our cohort. Taken together, there are several key differences between this study and prior studies on outcomes in pediatric MOGAD‐ON which ultimately make it challenging to compare our findings to previously published studies, but importantly, highlight how contemporary treatment protocols may shift the natural history of pediatric MOGAD‐ON toward a more favorable, monophasic course.

### 4.6 Limitations

There are multiple important and clinically relevant limitations to this study. First, this was a retrospective study with a limited sample size from a single center. Our cohort was small compared to previous multi‐center studies on MOGAD and ON but is likely the largest single‐center study on isolated optic neuritis in children, which allows for comparisons across relatively standardized protocols and data acquisition. Additionally, the center is a large tertiary‐quaternary referral center in the country's 5th‐most populous Metropolitan Statistical Area in the country [51], which introduces referral bias that may skew toward severe clinical courses and worse outcomes. Further, there was limited follow‐up and therefore limited data acquisition with some missing variables at some time points and limited duration since incident ON episodes. We relied on the Snellen test for visual acuity, which is a high‐contrast test. We do not have data on low‐contrast visual acuity, which prior reports suggest may be more affected by ON than is high‐contrast visual acuity [52]. We also do not have data on GCL thickness, and therefore relied on RNFL thickness as a surrogate marker of optic nerve pathology.

### 4.7 Future Directions

We aim to conduct longitudinal follow‐up to test the hypothesis of axonal reserve and long‐term visual acuity in adults who had ON as children. Additionally, future studies will attempt to integrate low‐contrast visual acuity and GCL thickness into our outcomes.

## 5 Conclusions

In the largest single‐center retrospective study of isolated pediatric optic neuritis, we found no children to later be diagnosed with MS. We demonstrate that MOGAD is the predominant etiology, occurring in over two‐thirds of cases. While all cases of isolated optic neuritis recovered excellent visual acuity despite reduced RNFL thickness, we found a linear relationship between the severity of high‐contrast visual acuity loss at the acute onset of ON and future thinning of the optic nerve. We provisionally hypothesize that this abrupt thinning of the optic nerve due to ON may accelerate age‐related visual decline in adulthood. Finally, one patient with MOGAD‐ON relapsed, considerably fewer than expected. However, multiple differences between our study design and application of clinical management versus prior studies may help to explain the less‐than‐expected rate of relapse.

## Author Contributions

Conception and design of the study: Chaitanya Aduru, Akansha Chandrasekar, Alexander J. Sandweiss. Acquisition and analysis of data: Chaitanya Aduru, Akansha Chandrasekar, Kyla Blasingame, Jonathan Rosen, Jesse Levine, Karla Salazar, Jonathan M. Yarimi, Alexander J. Sandweiss. Drafting of the manuscript and/or figures: Chaitanya Aduru, Akansha Chandrasekar, Kyla Blasingame, Jonathan Rosen, Jesse Levine, Karla Salazar, Madhuri Chilakapati, Rod Foroozan, Victoria Hardwick, Jonathan M. Yarimi, Nikita Shukla, Timothy E. Lotze, Kristen S. Fisher, Alexander J. Sandweiss.

## Funding

This work was supported by through support from the Research Vision at Texas Children's Hospital.

## Conflicts of Interest

The authors declare no conflicts of interest.

## Data Availability

The data that support the findings of this study are available on request from the corresponding author. The data are not publicly available due to privacy or ethical restrictions.
